# Neutral contrast MRI for the detection of peripheral arterial wall calcifications

**DOI:** 10.1186/1532-429X-16-S1-O76

**Published:** 2014-01-16

**Authors:** Robert R Edelman, Oisin Flanagan, Shivraman Giri, Ioannis Koktzoglou

**Affiliations:** 1 Radiology, NorthShore University HealthSystem, Evanston, Illinois, USA; 2 Siemens Healthcare, Chicago, Illinois, USA; 3 Radiology, Pritzker School of Medicine, University of Chicago, Chicago, Illinois, USA; 4 Radiology, Feinberg School of Medicine, Northwestern University, Chicago, Illinois, USA

## Background

Peripheral arterial disease (PAD) is a potentially debilitating condition affecting more than 200 million people worldwide. CT angiography (CTA) has become the preferred modality of vascular surgeons for the imaging evaluation of PAD, because of its simplicity and widespread availability. However, vessel wall calcifications, frequently present in diabetic and elderly patients, diminish its clinical utility (1). MR angiography (MRA) is also an accurate test for PAD (2). Unlike CTA, vessel wall calcifications are invisible with MRA and do not impair diagnostic accuracy. However, the invisibility of calcifications is also a substantial deficiency given that the distribution and severity of vessel wall calcifications have clinical implications for interventional treatment strategy (3), and the presence of vessel wall calcifications contributes additional risk for morbidity and mortality. In order to overcome this significant limitation, we have implemented a novel "neutral contrast 3D MRI" (NCMRI) technique that detects vessel wall calcifications with CT-like spatial resolution over large fields of view.

## Methods

Patients with PAD and arterial wall calcifications by CTA were imaged at 1.5 Tesla. NCMRI uses an optimal combination of echo spacing (15.5 ms), flip angle (10 degrees), and in-phase TE (9.53 ms with flow compensation) in order to make blood within the vessel lumen appear bright, muscle and fat appear gray, while calcifications appear dark. The use of flow compensation and an excitation flip angle near the Ernst angle of blood and muscle helps to avoid flow voids and partly accounts for the similar signal of muscle and fat. Scan times are on the order of 5-8 minutes. Images are viewed with an inverted gray scale, so that calcifications and bone cortex stand out as bright structures (like CTA), whereas muscle and fat remain a neutral gray and the vessel lumen appears gray or dark.

## Results

With NCMRI, background tissues appeared uniformly gray, which contrasted with dark calcifications and allowed for the creation of minimum intensity projections. With inversion of the gray scale, NCMRI provided an image appearance similar to CTA. The signal to noise ratios were: muscle = 45.6, fat = 44.3, right common femoral artery (CFA) calcification = 5.5, right CFA lumen = 76.5, left CFA plaque = 48.0. The contrast-to-noise ratios were muscle-fat = 1.3, calcification-muscle = -40.1, calcification-lumen = -71.0.

## Conclusions

Neutral contrast MRI provides, for the first time, an efficient, volumetric approach for the detection of peripheral vessel wall calcifications by MRI. 1. Ouwendijk R et al. Radiology. 2006 Nov;241(2):603-8. 2. Menke J, Larsen J. Ann Intern Med. 2010;153:325-334. 3. Pentecost MJ et al. Circulation. 1994; 89(1):511-531.

## Funding

NIH 1R01HL096916 and Siemens Healthcare.

**Figure 1:**
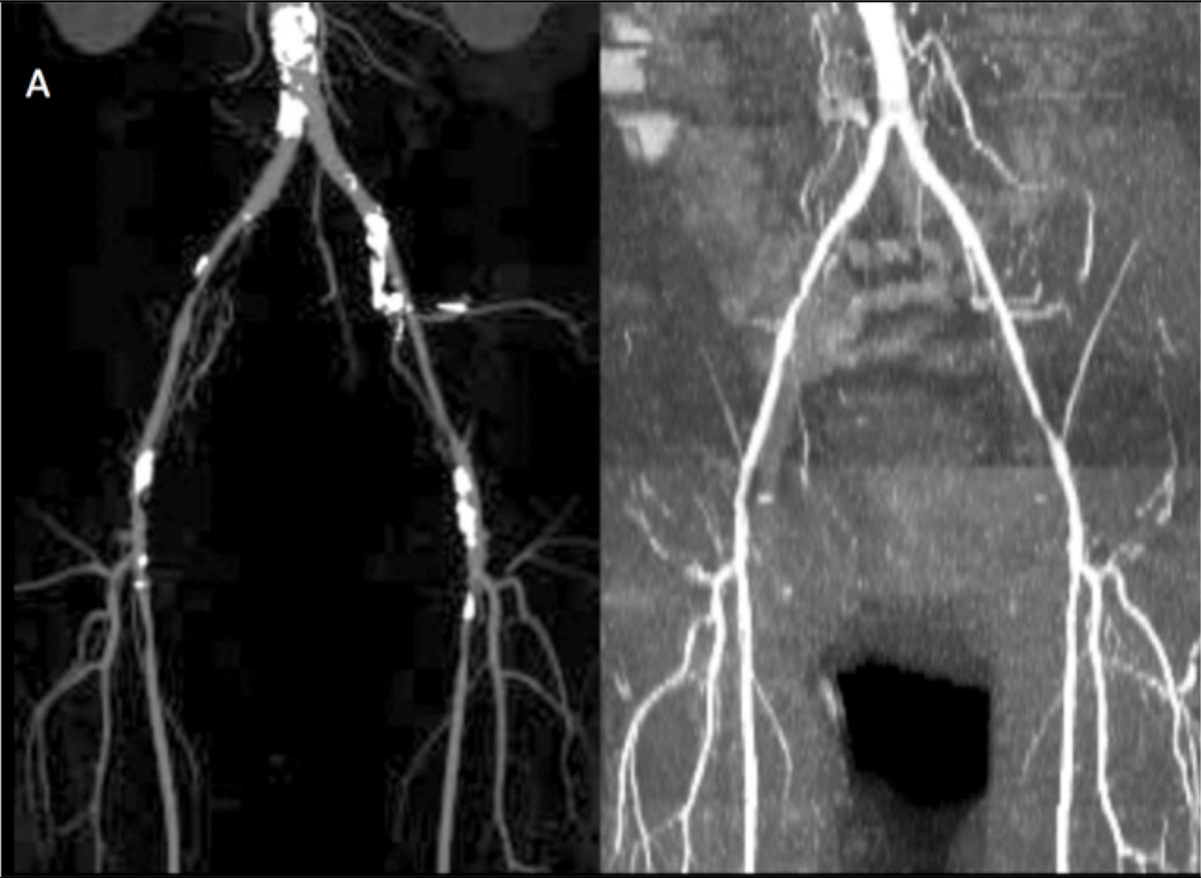
**60-year old male with PAD**. (A) MIP from a peripheral CTA (left) shows extensive vascular calcifications that partially obscure the arterial lumen, but are invisible in the MIP of the QISS MRA (right).

**Figure 2:**
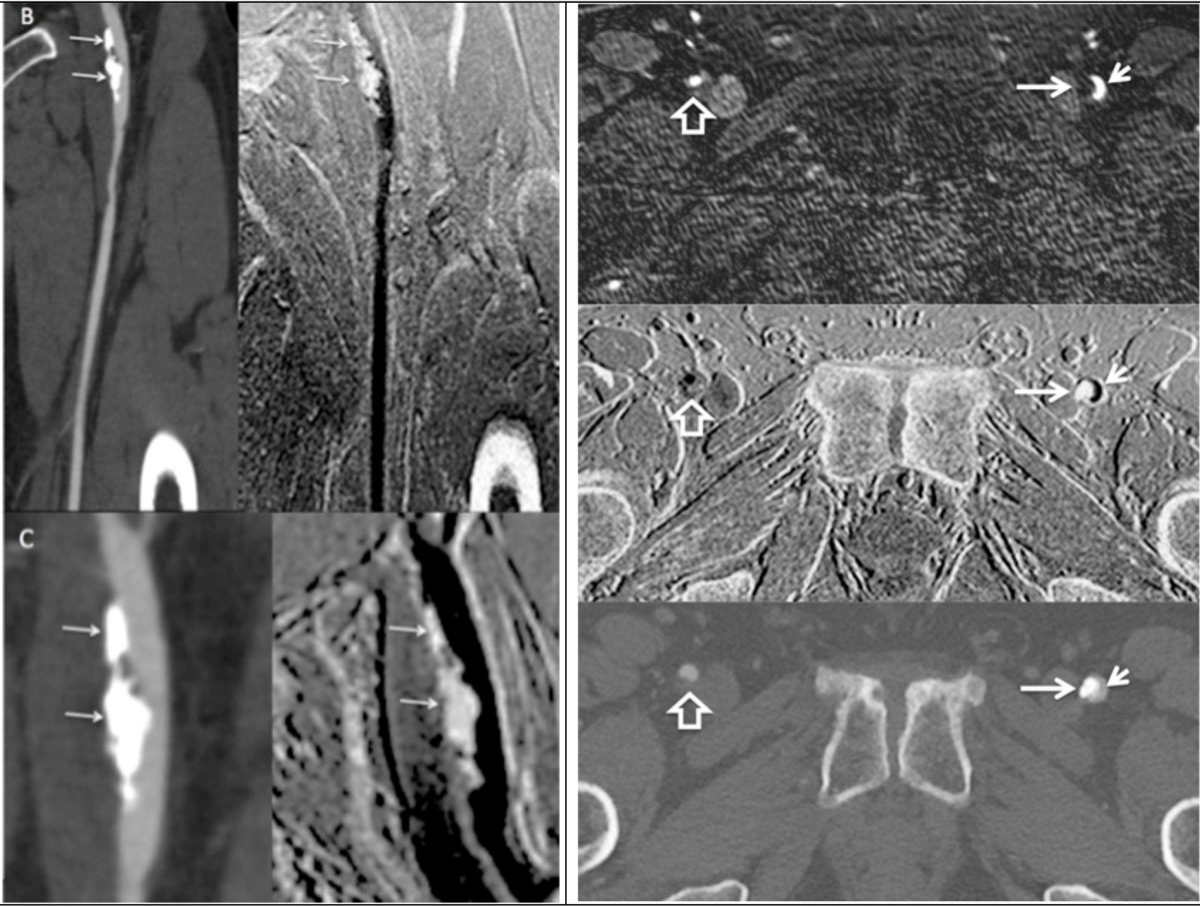
**(B) Comparison of curved MPR in the CTA (left) and curved minimum projection neutral contrast MRI (right)**. Wall calcifications (arrows) in the distal left common femoral artery (CFA) are comparably shown by the two modalities. (C) Magnified view again shows excellent correlation between the modalities. (D) Axial views, in order from the top: QISS MRA, neutral contrast MRI, CTA. Long arrow: left CFA wall calcifications; short arrow: left CFA lumen; open arrow: non-calcified plaque in right CFA.

